# A Functional Minigenome of Parvovirus B19

**DOI:** 10.3390/v14010084

**Published:** 2022-01-04

**Authors:** Alessandro Reggiani, Andrea Avati, Francesca Valenti, Erika Fasano, Gloria Bua, Elisabetta Manaresi, Giorgio Gallinella

**Affiliations:** Department of Pharmacy and Biotechnology, University of Bologna, 40138 Bologna, Italy; alessandro.reggiani5@unibo.it (A.R.); andrea.avati@student.unisi.it (A.A.); francesca.valenti@ior.it (F.V.); erika.fasano2@unibo.it (E.F.); gloria.bua2@unibo.it (G.B.); elisabetta.manaresi@unibo.it (E.M.)

**Keywords:** parvovirus B19, synthetic genome, genetic engineering, replicon unit, functional complementation

## Abstract

Parvovirus B19 (B19V) is a human pathogenic virus of clinical relevance, characterized by a selective tropism for erythroid progenitor cells in bone marrow. Relevant information on viral characteristics and lifecycle can be obtained from experiments involving engineered genetic systems in appropriate in vitro cellular models. Previously, a B19V genome of defined consensus sequence was designed, synthesized and cloned in a complete and functional form, able to replicate and produce infectious viral particles in a producer/amplifier cell system. Based on such a system, we have now designed and produced a derived B19V minigenome, reduced to a replicon unit. The genome terminal regions were maintained in a form able to sustain viral replication, while the internal region was clipped to include only the left-side genetic set, containing the coding sequence for the functional NS1 protein. Following transfection in UT7/EpoS1 cells, this minigenome still proved competent for replication, transcription and production of NS1 protein. Further, the B19V minigenome was able to complement B19-derived, NS1-defective genomes, restoring their ability to express viral capsid proteins. The B19V genome was thus engineered to yield a two-component system, with complementing functions, providing a valuable tool for studying viral expression and genetics, suitable to further engineering for purposes of translational research.

## 1. Introduction

Within the family *Parvoviridae* [1], Parvovirus B19 (B19V) is a human pathogenic virus of clinical relevance, responsible for transient or persistent erythroid aplasia, infectious erythema, arthropathies, myocarditis and intrauterine infections, among others [2,3]. The variability in the pathogenetic processes and the resulting clinical outcomes of diverse nature and severity depend on a complex interplay between the viral properties, the characteristics of target cells in the different tissues, and the physiological status and immune response of infected individuals. B19V has a marked tropism for erythroid progenitor cells (EPCs) in the bone marrow, both susceptible and permissive depending on their differentiation and proliferation state [4]. In EPCs, infection normally induces cell cycle arrest and apoptosis [5], thus causing a temporary block in erythropoiesis which can become clinically relevant [6]. Different non-erythroid cell types, including endothelial, stromal, or synovial cells, are also susceptible but mainly non-permissive. In these cells, infection can trigger inflammatory responses and consequent tissue damage [7], and generally results in long-term persistence of viral DNA within tissues [8].

Investigation of viral genetics is fundamental to better understanding of the B19V replication cycle, the virus–cell interaction in different environments, the pathogenetic processes underlying the wide range of associated diseases, and the devising of more efficient antiviral strategies [9,10]. The B19V genome is composed of two inverted, terminal repeated regions (ITR) of imperfect palindromic sequence, 383 nt long, flanking a unique internal region (IR), 4830 nt long, containing all the coding sequences (Figure S1). The role of ITRs is crucial. The ability of palindromic sequences to fold in self-priming, hairpin secondary structures, and the presence of specific cis-recognition sequences acting as origins of replication, allow replication of the viral DNA through a rolling hairpin mechanism [11,12,13]. The activity of the unique transcription promoter (P_6_) at the left end of the internal region also depends critically on regulatory sequences within the upstream ITR [14,15,16,17,18]. Within the IR, distribution of splicing and cleavage-polyadenylation recognition sequences along the genome ensures coordinate processing of the pre-mRNA to a set of mature mRNAs [19,20,21,22]. Functionally, these can be divided into a set expressed from the left side of the genome, mainly coding for NS1 protein in an early phase of replication, and a set expressed from the right side of the genome, mainly coding for the structural VP proteins in the late phase of replication [23,24,25].

NS1 protein is the major non-structural protein, essential to virus replication and central for interaction with host cell components [9]. It is involved in the replication of the B19V genome, by its capacity to bind to specific recognition sites in the terminal regions, and by its endonuclease and helicase activities effecting ITR terminal resolution and strand unwinding. It is involved in viral transcription, enhancing activity of the P_6_ promoter by its trans-activating domains, thus promoting overall B19V genome expression. Besides, NS1 is a heterologous trans-activator of cellular genes, therefore inducing alterations in the cellular environment. It has a role in regulating progression through the cell cycle and in inducing apoptosis, therefore contributing to the pathogenesis of B19V infection. Given all this, NS1 is a matter of relevant interest in studying B19V, not least as a pharmacological target [26,27]. Viral capsid proteins VP1 and VP2 assemble to form a capsid shell of 22 nm in diameter, arranged in a T = 1 icosahedral structure [28]. Being translated from the same coding frame, both proteins share a common region that forms the core shell, while the VP1 protein, about 5% in abundance, possesses an additional N-terminal region, VP1u, crucial for cell recognition, attachment and penetration [29,30].

Information can be obtained from experiments involving engineered genetic systems in appropriate in vitro cellular models, since cloned forms of the B19V genome can be competent for replication and constitute an effective tool for studying the viral lifecycle and its interaction with target cells [31,32]. Previously, a model system was established by a novel synthetic strategy [33]. A reference genome of defined consensus sequence was designed, synthesized and cloned in a complete and functional form in a plasmid vector. Such a genome was able to replicate and produce infectious viral particles in a producer/amplifier cell system, the myeloblastoid UT7/EpoS1cells, allowing generation and further propagation of virus in EPC cell cultures. Replicative competence was linked to preservation of sequence integrity and asymmetry within the terminal regions, while preservation of the complete internal region ensured maintenance of the cis-acting signals required for regulation of viral genome expression. Therefore, the full proteome of B19V could be co-ordinately expressed and novel infectious viral particles produced. Further investigation is required to assess the flexibility of this system to genetic manipulation, and its potential as an advanced tool for basic research and translational applications.

With this aim, the main objective of the present experiments was to create a genetic unit derived from the complete, competent cloned B19V genome, simplified to a potential replicon unit. In this minigenome, the terminal regions were maintained in a form able to sustain viral replication, while the internal region was clipped to contain only the left-side genetic set. This would only allow the production of the subset of mRNAs corresponding to the early phase, including the mRNAs for NS1 protein. Following transfection in UT7/EpoS1 cells, replication, transcription and production of NS1 protein were monitored to assess the functional competence of the minigenome. Further, the capacity of this minigenome to complement defective forms of B19 genome rescuing production of viral capsid proteins and the possibility of producing transducing viral particles was also tested.

## 2. Materials and Methods

### 2.1. Molecular Cloning

Experiments were carried out on the previously established pCK10 and pCH10 plasmid clones containing the reference B19V EC sequence (GenBank KY940273) [33]. Plasmids pCK10 and pCH10 contain as inserts B19V EC segments, including the complete internal region and extension of both ITRs beyond the sites of dyad symmetry (pCK10, nt. 136–5461) or up to the sites of dyad symmetry (pCH10, nt. 184–5413).

The derived pCK10-pAs1 and pCH10-pAs1 plasmids were obtained by deletion of the genomic segment between nt. 2813–5169. To the purpose, a synthetic segment of appropriate sequence was transferred into pCK10 and pCH10 to replace the original insert by cloning using the XmaI and BssHII sites at positions 2251 and 5413. The derived pCH10-A1.1 and pCH10-A1.2 plasmids were obtained by deletion of the genomic segments between nt. 588–2089 and 588–2209, respectively. Synthetic segments of appropriate sequence were transferred into pCH10 to replace the original insert by cloning using the BssHII sites and BamHI sites at positions 184 and 4076.

Synthetic DNA inserts were obtained from Eurofins Genomics. Restriction endonuclease (RE) and ligase enzymes were obtained from Thermo Fisher Scientific and used according to manufacturer’s directions. Plasmid clones were maintained in SURE bacterial cells (Agilent Technologies, Santa Clara, CA, USA) under ampicillin selection and growth in LB medium at 30 °C. Plasmid DNA purification was performed by PureYield Plasmid Midiprep (Promega, Madison, WI, USA). Inserts used for transfection assay were amplified by PCR by using the Expand High Fidelity System (Roche, Basel, Switzerland) as described, further purified by using Wizard SV Gel and PCR clean-up system (Promega) and quantified by UV absorbance determination.

### 2.2. Cell Culture

UT7/EpoS1 cells, obtained from KE Brown [34], were cultured in IMDM (Cambrex, East Rutherford, NJ, USA), 10% FCS and 2 U/mL Epo α (Eprex, Janssen, Beerse, Belgium), at 37 °C and 5% CO_2_. Cells were kept in culture at densities between 2 × 10^5^–1 × 10^6^ cells/mL and used for transfection experiments when at a density of 3 × 10^5^ cells/mL. Erythroid progenitor cells (EPCs) were generated in vitro from peripheral blood mononuclear cells (PBMC) obtained from the leukocyte-enriched buffy coats of healthy blood donors, from the Immunohematology and Transfusion Service, S. Orsola-Malpighi University Hospital, Bologna (http://www.aosp.bo.it/content/immunoematologia-e-trasfusionale; authorization 0070755/1980/2014, issued by Head of Service). Availability was granted under conditions complying with Italian privacy law. Neither specific ethics committee approval nor written consent from donors was required for this research project. In vitro culture was carried out following established protocol [35], and cells were used for infection experiments at day 8 of in vitro growth and differentiation.

### 2.3. Transfection and Infection

UT7/EpoS1 cells were transfected by using the Amaxa Nucleofection System (Lonza, Basel, Switzerland), with V Nucleofector Reagent and T20 program setting, at a ratio of 1 µg insert DNA for 10^6^ cells. Following transfection, the cells were incubated at 37 °C and 5% CO_2_ in complete medium at an initial density of 10^6^ cells/mL, until collection at the indicated time points. Cells and cell-free supernatants were separated by centrifugation at 4000 rpm for 5 min in microfuge (Eppendorf, Hamburg, Germany), then fractions were used for analysis and/or successive infection experiments.

For infection experiments, cell-free supernatants obtained from transfected UT7/EpoS1 cells were added to EPCs cells at a ratio of 100 µL for 1 × 10^6^ cells. Infection was carried out at 37 °C for 2 h, then cells were washed free of inoculum and expanded in complete medium at 37 °C and 5% CO_2_ at an initial density of 10^6^ cells/mL, until collection at the indicated time points and subsequent processing as described.

### 2.4. Quantitative Molecular Analysis

Experimental samples were processed for total nucleic acid purification by using the Viral Total Nucleic Acid kit for the Maxwell 16 extractor (Promega), then quantitative determination of B19V nucleic acids (viral DNA, total mRNA, mRNA subsets) was carried out by qPCR and qRT-PCR according to previously established protocols [23,24]. Genomic DNA coding for 18S rRNA (rDNA) was amplified for calibration with respect to cell copy number. Absolute quantification of both viral DNA and total viral RNA was obtained by using the primer pair R2210–R2355, located in the central exon of B19V genome, while determination of the relative abundance of the different subsets of viral transcripts was obtained by using a selected array of primer pairs, as indicated in Table 1. For the DpnI Assay, DNA previously treated with either EcoRI or EcoRI+DpnI restriction enzymes was amplified by using primers encompassing DpnI site at position 1801 on the B19V genome, and the fraction of DNA not cleaved by the enzyme determined by qPCR analysis and absolute quantitation with respect to an external calibration curve.

### 2.5. IIF and Cytofluorimetric Analysis

For detection of viral proteins by immunofluorescence, aliquots of 5 × 10^4^ cells were spotted on glass slides and fixed with 1:1 acetone:methanol for 10 min at −20 °C. For detection of NS protein, cells were incubated with the human monoclonal antibody MAb1424 (kindly supplied by Susanne Modrow) (1:100 in PBS/FCS 10%), then with an anti-human FITC-conjugated secondary antibody (Dako, 1:20 in PBS/FCS 10%). For detection of VP proteins, cells were incubated with a monoclonal mouse antibody against VP1 and VP2 proteins (MAb8293, Chemicon, Merck Millipore, Milan, Italy) (1:200 in PBS/BSA 1%), then with AlexaFluor488 anti-mouse secondary antibodies (Life Technologies, Monza, Italy) (1:1000 in PBS/BSA 1%). Cell populations were also analysed for expression of viral proteins by using flow cytometry (FACSCalibur, Becton Dickinson, Milan, Italy). Aliquots of 10^6^ cells were fixed in PBS/formaldehyde 0.5% O/N at 4 °C, permeabilized in PBS/saponin 0.2% at RT while rocking for 45 min and incubated in suspension with antibodies diluted in PBS/FCS 2% (1:100 NS primary; 1:40 anti-human FITC secondary). Data were analysed using the Cell Quest Pro Software (Becton Dickinson).

## 3. Results

### 3.1. Design and Construction of a B19V Minigenome

**Figure 1:**
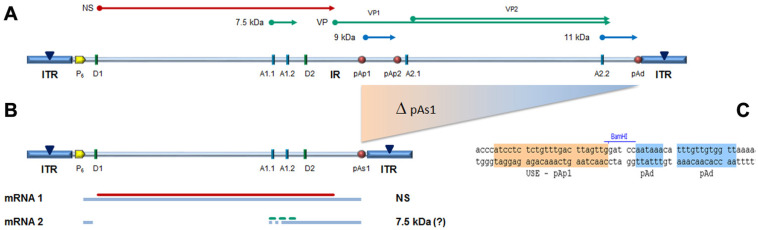
(**A**) Map of B19V genome. ITR: inverted terminal repeats (▼, site of dyad symmetry). IR: internal region and relevant cis-acting functional sites (P_6_, promoter; pAp1, pAp2, proximal cleavage-polyadenylation sites; pAd, distal cleavage-polyadenylation site; D1, D2, splice donor sites; A1.1, A1.2, A2.1, A2.2, splice acceptor sites). Coding sequences for viral NS, VP and smaller non-structural proteins are aligned to map. **Δ**: deletion to create a novel cleavage-polyadenylation signal (pAs1). (**B**) Map of B19V derived minigenome; simplified transcription map, indicating the two classes of mRNAs (mRNA 1–2), with alternative splicing forms (dashed lines) and related coding potential. (**C**) Sequence at the novel pAs1 cleavage-polyadenylation site (USE—pAp1, upstream element to pAp1 [19]).

In the design of a B19V minigenome with potential replicative activity, the rational requirements were: (i) to preserve both terminal regions up to the sites of dyad symmetry, retaining the capacity of hairpin formation; and (ii) to preserve the internal region extending up to the pAp1 proximal cleavage-polyadenylation signal, as a gene cassette with potential for coding for the NS1 protein, while eliminating the genomic region coding for the viral capsid proteins. To this end, a large deletion was operated in the right-side of genome, and a novel chimeric cleavage-polyadenylation signal created, named pAs1, joining the upstream cis-elements of pAp1 and the downstream cis-elements of pAd (Figure 1).

For the construction of the minigenome, a synthetic gene segment encompassing the designed deletion substituted by the novel pAs1 sequence was synthesised and inserted in order to replace the original sequence in the previously established pCK10 and pCH10 plasmids [33]. The viral insert in pCK10 preserves both terminal regions extending beyond the site of dyad symmetry; however, attempts at cloning in pCK10 only yielded unstable plasmid clones, with deletion of the palindromic sequence in the right-hand terminal region. The viral insert in pCH10 preserves both terminal regions extending up to the site of dyad symmetry; in this case, a stable plasmid clone was successfully obtained, named pCH10-pAs1.

### 3.2. Functional Competence of the B19V Minigenome

**Figure 2:**
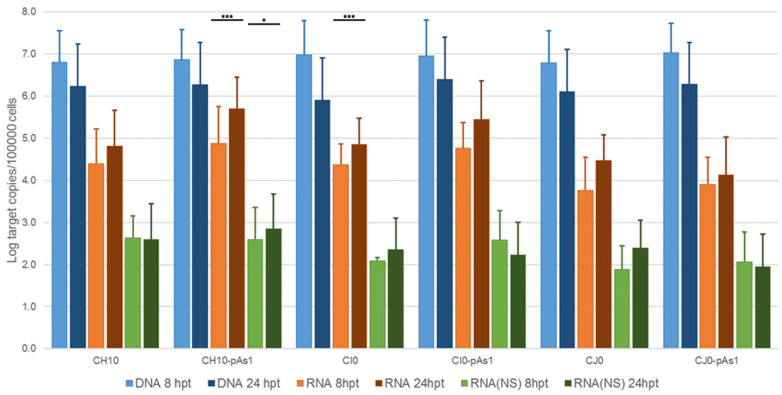
Viral nucleic acids in UT7/EpoS1 cells, transfected with CH10 and CH10-pAs1 derived inserts. Log amounts of target copies (viral DNA, total RNA, NS1 mRNA), normalized to 10^5^ cells, at 8 and 24 hpt. Mean and std of duplicate determinations for two different experiments. Two-way ANOVA, Bonferroni post-test: ***, *p* < 0.001; *, *p* < 0.05.

From the pCH10 plasmid, three genomic inserts of different extension can be obtained, differing in their functional competence: CH10, corresponding to the whole cloned insert, extending in the terminal regions to the sites of dyad symmetry (nt. 184–5413); CI0, extending in the terminal regions within the sites of dyad symmetry (nt. 245–5474); and CJ0, extending in the terminal regions only to the start of palindromes (nt. 366–5231). From the pCH10-pAs1 plasmid, three genomic inserts of corresponding extension could also be obtained by analogy: CH10-pAs1, CI0-pAs1, and CJ0-pAs1. The biological activity and functional competence of pCH10 and pCH10-pAs1 derived inserts was comparatively analysed following transfection in UT7/EpoS1 cells. Genomic inserts were obtained by means of in vitro amplification, then purified inserts were used to transfect UT7/EpoS1 cells. At 8- and 24-h post-transfection (hpt), aliquots of cell culture were sampled for quantification of viral nucleic acids (DNA, mRNAs) by qPCR and qRT-PCR (Figure 2, Table S1A), and detection of the NS protein by IIF and cytofluorimetric analysis.

The amount of DNA, either at 8 or 24 hpt, was comparable for all tested inserts, indicating a similar transfection efficiency. Due to the large quantity of input DNA used in transfection, no significant temporal variation in DNA amount was observed for any of the tested inserts, apart from a general decrease from 8 to 24 hpt (mean 0.70 Log, range −1.1–0.57), likely to be due to progressive degradation of exogenous DNA. De novo synthesis of transfected DNA was assessed in a parallel experiment, by transfection of inserts directly excised from plasmids and tested for *Dam* methylation pattern and resistance to DpnI cleavage, both by a qPCR assay and a Southern Blot analysis. In this way, it was possible to investigate CH10 and CH10-pAs1, excised using BssHII, CI0 and CI0-pAs1, excised using AccIII, but not CJ0 and CJ0-pAs1, because of lack of corresponding RE sites.

**Figure 3:**
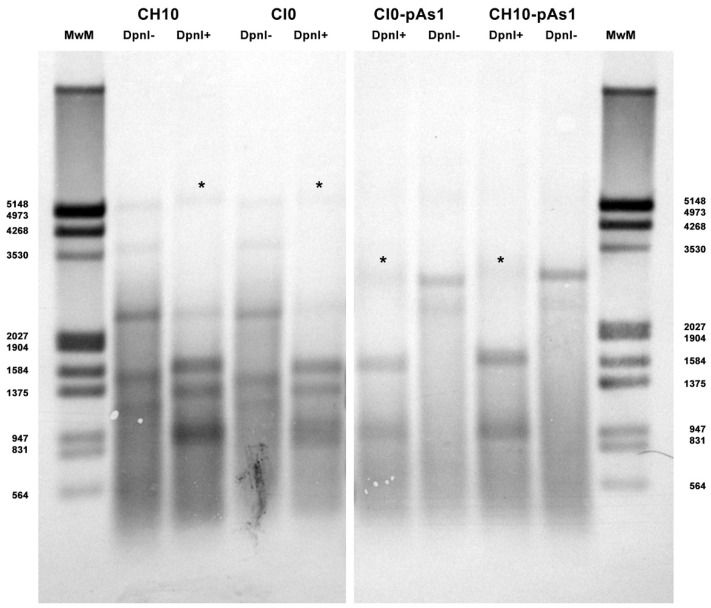
Southern Blot Analysis of B19V DNA obtained from UT7/EpoS1 cells transfected with inserts CH10, CI0 and CH10-pAs1, CI0-pAs1, collected at 24 hpt. Samples were treated by RE DpnI to distinguish de novo synthesized viral DNA (*) based on different *dam* methylation pattern and sensitivity to RE DpnI. MwM: molecular weight marker III, Dig-labelled (Roche). Southern Blotting and hybridization using a full-length digoxigenin-labelled DNA probe was carried out as described [33].

By qPCR, DNA excised from plasmids was resistant to 1.7% and 1.4% for CH10 and CH10-pAs1, and 1.6% and 1.3% for CI0 and CI0-pAs1. DNA obtained from transfected cells at 24 hpt was resistant to 11.7% and 15.8%, and 8.8% and 19.0%, respectively. By Southern Blot (Figure 3), at 24 hpt, bands corresponding to full-length, DpnI resistant DNA were also observed for all transfected inserts. Data thus obtained are consistent with the maintenance of the replicative competence of transfected inserts, at least for CH10-and CI0-derived inserts, both the complete genomes and the derived minigenomes, a property likely to be due to the preservation of sequence symmetry within the terminal regions and implying a hairpin-independent priming of DNA synthesis.

All transfected inserts showed a sustained transcriptional activity. Viral mRNAs were detected for all inserts at both time-points post-transfection, with a general increase from 8 to 24 hpt of total mRNA (mean 0.56 Log, range −0.01–1.33), and to a lesser extent of NS mRNA (mean 0.08 Log, range −0.36–0.51), significant for CH10-pAs1 only. Although with some variability, overall results attested the early onset and maintenance of viral transcription, implying processing of pre-mRNA at the novel chimeric pAs1 cleavage-polyadenylation site, and preservation of a balanced usage of splicing signals. In fact, a typical ratio of mRNAs pertaining to the left-side genome was produced, including both the unspliced mRNAs coding for NS protein (mRNA 1 in Figure 1), approximately 1% of total viral mRNAs, and the more abundant spliced mRNAs (mRNA 2 in Figure 1), in a pattern analogous to that observed in the early phase of the replicative cycle.

**Figure 4:**
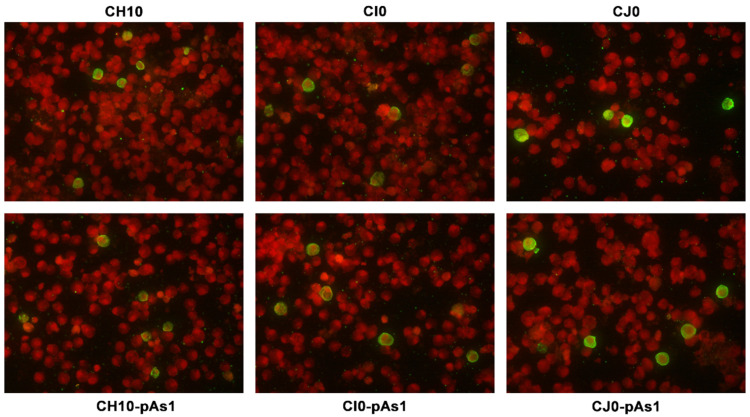
UT7/EpoS1 cells transfected with the indicated inserts were sampled at 24 hpt, and NS1 protein was detected by IIF. Original magnification 400×.

The expression of NS protein was monitored by IIF and cytofluorimetric analysis, sampling transfected cell cultures at 8 and 24 hpt. By IIF (Figure 4), NS protein was already observed at 8 hpt, and at 24 hpt the number of positive cells and signal intensity both increased. Distribution of the protein within the cells showed the same nuclear/cytoplasmic pattern both for all complete or derived minigenome inserts.

**Figure 5:**
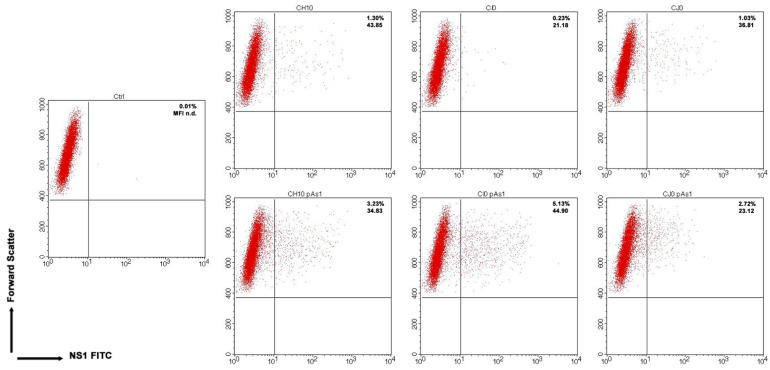
UT7/EpoS1 cells transfected with the indicated inserts (Ctrl, no DNA control) were sampled at 24 hpt, and cell population was analysed by cytofluorimeter to determine the percentage of NS1 expressing cells. Dot plot graph on gated cell population for FSc and NS1 FITC. Reported percentage values of positive cells and geometric mean fluorescence intensity (MFI) for positive subpopulations reported as the average result of two independent determinations.

By cytofluorimetric analysis (Figure 5), a quantitative assessment of cells expressing NS1 protein was obtained at 24 hpt. The percentage of positive cells was in the range 0.2–1.3% for the complete inserts and increased to 2.6–5.0% for the derived minigenomes, the highest increase was observed for the CI0/CI0-pAs1 combination. Altogether, experiments suggest that the lower genetic complexity of modified genomes promoted progressive expression and accumulation of NS protein in an increasing fraction of the cell population.

### 3.3. Functional Complementation of the B19V Minigenome

**Figure 6:**
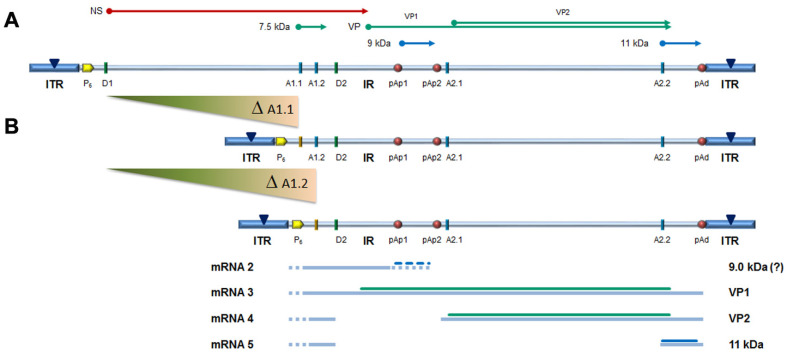
(**A**) Map of B19V genome, see Figure 1. **Δ**: deletion to remove first intron (A1.1/2). (**B**) Map of B19V derived minigenomes; simplified transcription map, indicating the four classes of mRNAs (mRNA 2–5), with alternative splicing/cleavage forms (dashed lines) and related coding potential.

The capacity of the CH10-pAs1 minigenome to provide complementing functions through expression of NS1 protein was thereafter tested in a subsequent series of experiments. To the purpose, a set of modified B19V genome clones was designed, defective for the coding sequence of NS protein. In particular, the pCH10 clone was modified by deletion, removing the genomic region corresponding to the first intron within the NS gene, from the splice donor site D1 to the two possible alternative splice acceptor sites A1.1 and A1.2. The obtained plasmids, named CH10-A1.1 and CH10-A1.2, retained the cis-acting sequences directing alternative cleavage-polyadenylation at pAp and pAd sites, sequences regulating alternative splicing of the distal introns at D2 and A2.1/2 sites, and all of the coding sequences for the VP1, VP2 and 11 kDa proteins (Figure 6).

**Figure 7:**
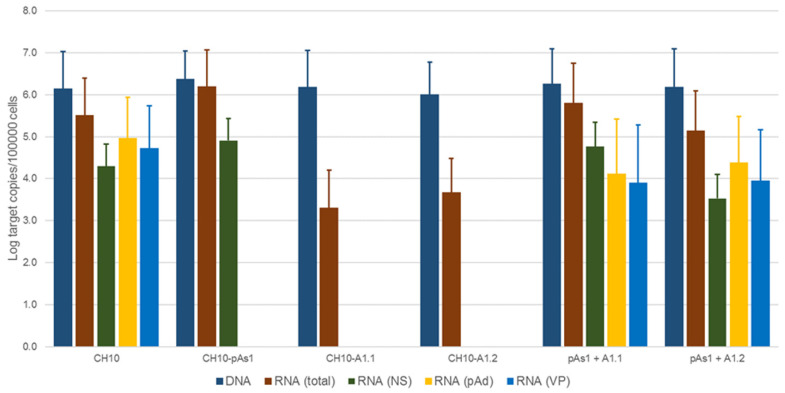
Viral nucleic acids in UT7/EpoS1 cells, transfected/co-transfected with CH10, CH10-pAs1 and CH10-A1.1/2 inserts. Log amounts of target copies (viral DNA, total RNA, NS1 mRNA, pAd cleaved RNA, VP RNA), normalized to 10^5^ cells, at 24 hpt. Mean and std of duplicate determinations for two different experiments.

To evaluate the functional competence and possible complementation effects for these defective genomes, inserts obtained by means of in vitro amplification were transfected in UT7/EpoS1 cells, alone or in co-transfection with CH10-pAs1 as helper plasmid. At 24 hpt, aliquots of cell culture were sampled for quantification of viral nucleic acids (DNA, mRNAs) by qPCR and qRT-PCR (Figure 7, Table S1B), and detection of the NS1 and VP proteins by IIF.

No significant differences were observed in the amounts of viral DNA, but relevant information was obtained by quantification of viral RNA. By comparison to the reference CH10 insert, insert CH10-pAs1 confirmed its high transcriptional activity and correct mRNA processing, with an abundance of about 1% unspliced mRNAs coding for NS protein. The NS defective, CH10-A1.1 and -A.2 inserts showed a reduced (−3 Log) basal transcriptional activity, which allowed detection of only the proximally cleaved mRNAs (mRNA 2 in Figure 6), and not of any distally cleaved mRNA (mRNAs 3–5 in Figure 5). Cotransfection of these inserts with CH10-pAs1 inset led to functional complementation, restoring expression from the NS-defective genomes, as shown by the detection of A1.1 and A.2 derived mRNAs, to amounts only about 1 Log lower than what observed for the reference CH10 insert. By composition, the most abundant mRNAs were still the proximally cleaved mRNA species (mRNA 2), which were contributed by both CH10-pAs1 (following splicing) and A1.1/2 (following cleavage at pAp); abundance of NS mRNA (mRNA 1), contributed only by CH10-pAs1, pAd cleaved mRNAs (mRNA 3–5) and VP mRNAs (mRNA 3–4), contributed only by A1.1/2, were in all cases in the order of 1% of total mRNAs.

**Figure 8:**
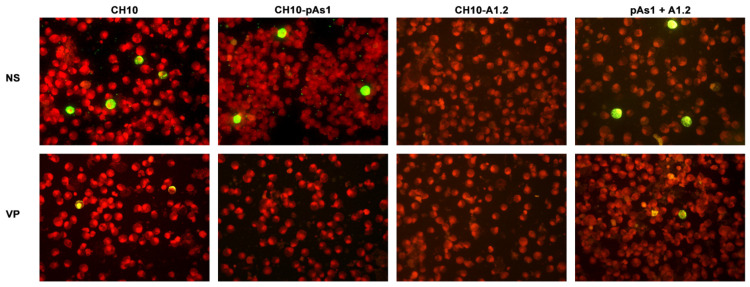
UT7/EpoS1 cells transfected with the indicated inserts were sampled at 24 hpt, and NS1 or VP1/2 proteins were detected by IIF. Results obtained for insert CH10-A1.1 were analogous to what obtained for CH10-A1.2 (not in figure). Original magnification 400×.

By IIF analysis (Figure 8), expression of both NS and to lesser extents VP proteins was confirmed for the CH10 insert. The expression of NS protein was also confirmed for CH10-pAs1, and not detected in the case of CH10-A1.1/2, as expected. Expression of VP proteins from CH10-A1.1/2 inserts alone was not observed. Cotransfection of CH10-pAs1 and A1.1/2 preserved expression of NS protein from CH10-pAs1 and restored the expression of VP proteins from CH10-A1.1/2, although for the latter detection was limited to a small number of cells. Altogether, data confirm that the NS protein produced by the CH10-pAs1 minigenome is functional in complementation of defective B19V genomes, restoring expression of the late set of mRNAs to a pattern similar to the standard expression profile of B19V genome and allowing production of capsid proteins.

### 3.4. Extracellular Vehiculation of Minigenomes

Following transfection of UT7/EpoS1 cells, measurable amounts of viral DNA were detectable in the cell culture medium until 6 days post-transfection, not associated to cells but partially resistant to nuclease treatment. The possibility that this genetic material could be transferrable to susceptible EPCs was investigated. For the purpose, CH10-pAs1 and CH10-A1.1/2 inserts were transfected in UT7/EpoS1 cells in the different combinations as described, and expression of NS and VP proteins first confirmed for all competent combinations. Then, after a 6 day course, the supernatant of transfected cell cultures was collected and added to in vitro differentiated EPCs cells, as a test system. After a further 48 h course of incubation, EPCs were collected and analysed for any presence of B19V DNA, RNA or expression of NS protein. For all experimental samples, a low measurable amount of DNA was found associated to EPCs (<10^2^ copies/10^5^ cells). However, no transcriptional activity could be detected in EPCs in any tested combination and no NS protein production could be observed. The obtained results imply that the vehiculation of genetic material from cells transfected with the CH10 derived clones may not be due to the formation of transducing viral particles, and that the process is not functionally relevant to a measurable extent. The hypothesis that such transfer should be attributed to simple carry-over in a nuclease-resistance form, or to the formation of extracellular vesicles or exosomes, and whether a limited number of transducing viral particles is actually produced, requires further investigation.

## 4. Discussion

In our present work, we designed and produced a B19V minigenome, derived from the complete, competent cloned B19V genome, simplified to a replicon unit. Following transfection in the UT7/EpoS1 cells, this minigenome still proved competent for replication, transcription and production of NS protein. Further, the B19V minigenome was able to complement B19-derived, NS-defective genomes, restoring their ability to express capsid proteins. The unique B19V genome was thus engineered to yield a two-component system, an element expressing the functional NS1 protein, the other the structural capsid proteins, with complementing functions.

In all engineered genetic elements, the terminal regions have been preserved up to the site of dyad symmetry and in opposite flip/flop configurations, thus meeting requirements for maintenance of replicative competence [12,33]. To this respect, interesting information has been obtained by comparing the activity of genomic inserts of different extension within the ITRs. De novo synthesis of transfected DNA could be demonstrated, as previously [33], for the complete CH10 and CI0 inserts, and this property was also conserved for the derived -pAs1 inserts. Hairpin-independent priming of DNA synthesis had been documented in a different experimental setting [11], and it is likely involved here.

The unique P_6_ promoter is present in the same position and pattern in all engineered clones, whereas the level of transcription of each one depends on the actual expression of NS protein, due to its strong trans-activating activity on its own promoter [14]. Transcription from the complete CH10, CI0, CJ0, and the derived -pAs1 inserts is detected at high levels already at an early time point (8 hpt), further increasing at a late time point (24 hpt). Transcription levels from the derived -pAs1 inserts is higher than the respective complete clones, but effects depending on the ITR extension are relatively minor. Conversely, sustained transcription from CH10-A.1/2 clones is detectable only upon complementation, and at the late time point.

CH10-pAs1 elements are simplified with respect to pre-mRNA processing. Reduction in the size and complexity of the transcriptional template and the introduction of a novel single cleavage-polyadenylation site at the end of the genome abrogates the early-late transcriptional switch typical of B19V, thus leading to sole accumulation of left-side cassette mRNAs [19,20]. Within these processes, retention of unaltered splicing signals ensures correct processing of pre-mRNAs and unaltered balance of unspliced, NS encoding to spliced mRNAs [25]. Thus, the net effect compared to that observed for complete clones is a sustained accumulation of NS-coding transcripts and overexpression of NS protein in a larger proportion of transfected cells.

Modifications introduced to obtain the defective CH10-A1.1/2 elements have different consequences. Reduction of the genetic template with deletion of the large left-side intron results in the absence of any NS coding mRNA, but both original cleavage-polyadenylation sites are maintained, therefore preserving a possible early-late switch in expression pattern. Moreover, the splicing signals maintained in the right gene cassette still allow alternative splicing events leading to the production of VP1, VP2 and 11 kDa encoding mRNAs [21,22]. In fact, experiments confirmed that such an mRNA set is produced when in the presence of complementing NS protein. However, compared to the complete genome, a relatively higher proportion of short (proximally cleaved, spliced) mRNAs is produced, likely because of the contribution from both transcriptional templates and prevalent pre-mRNA processing. As a consequence, the amount of VP-encoding mRNA obtained from CH10-A.1/2 templates is reduced to suboptimal levels, although enough to achieve production of VP proteins.

The present work reached relevant goals, while showing some critical limitations inherent to the system. A minigenome with characteristics of a replicon has been constructed, able to replicate and overexpress NS protein. Such minigenome may constitute a valuable tool for studying the function of NS protein within a simplified viral genetic contest, mainly its impact on cell functionality, or in the search of molecules with antiviral activity. A functional complementation between defective genomes was obtained, since the minigenome restored the ability of separate genetic units to express capsid proteins. This property opens the possibility of engineering the B19V genome with less stringent structural constraints, allowing for example to modify cis-acting genomic elements, mutagenize or add expression tags to the sequences coding for proteins, or insert heterologous reporter genes, to the purpose of a deeper characterization of B19V replication, expression and interaction in the cellular environment.

Ideally, the compresence in a same cell of two genetic units with complementing functions opens a possibility for packaging and generation of transducing viral particles. However, generation of transducing virus was not shown in our experiments, thus constituting the major limit of the present work. All transfection experiments have been conducted in the UT7/EpoS1 cell line, which is appropriate for research on B19V [36], but at the expense of a very low transfection efficiency. When transfected with a complete genomic insert, de novo produced virus can be subsequently amplified in primary EPCs to yield infectious virus at high titre. In the case of cotransfection of separate complementing units, transducing viral particles would not benefit of any possible subsequent amplification passage. While generation of engineered virus specifically targeting a selected cell population such as EPCs would be of translational interest, further intense research is required to achieve such goal.

## Figures and Tables

**Table 1:** Primer combinations used in the qPCR and qRT-PCR assays for the detection and quantitative evaluation of B19V nucleic acids. See also Figure S1 for primer location on B19V genome.

Primer	Sense	Primer	Antisense	DNA Target
18Sfor	CGGACAGGATTGACAGATTG	18Srev	TGCCAGAGTCTCGTTCGTTA	Genomic 18S rDNA
R2210	CGCCTGGAACACTGAAACCC	R2355	GAAACTGGTCTGCCAAAGGT	Virus DNA
D1801f	CTTGGTGGTCTGGGATGAAG	D1801r	TACTCCAGGCACAGCTACAC	for DpnI Assay
**Primer**	**Sense**	**Primer**	**Antisense**	**RNA Target**
R1882	GCGGGAACACTACAACAACT	R2033	GTCCCAGCTTTGTGCATTAC	NS mRNA
R2210	CGCCTGGAACACTGAAACCC	R2355	GAAACTGGTCTGCCAAAGGT	Central exon, total RNA
R4869	ATATGACCCCACAGCTACAG	R5014	TGGGCGTTTAGTTACGCATC	VP1/2 mRNA
R4899	ACACCACAGGCATGGATACG	R5014	TGGGCGTTTAGTTACGCATC	Distal exon, pAd cleaved

## Supplementary Materials

The following are available online at https://www.mdpi.com/article/10.3390/v14010084/s1, Figure S1: B19V genome organization, transcription map and primer location. Table S1: Quantitation of viral nucleic acids.
