# Supplementary material for: A Functional Minigenome of Parvovirus B19
Source: Viruses. 2022 Jan 4;14(1):84. doi: 10.3390/v14010084 (PMC8780457; doi:10.3390/v14010084)
Supplement: Supplementary file 1 [file viruses-14-00084-s001.zip › viruses-1519880-supplementary.pdf]

Figure S1. B19V genome organization, transcription map and primer location

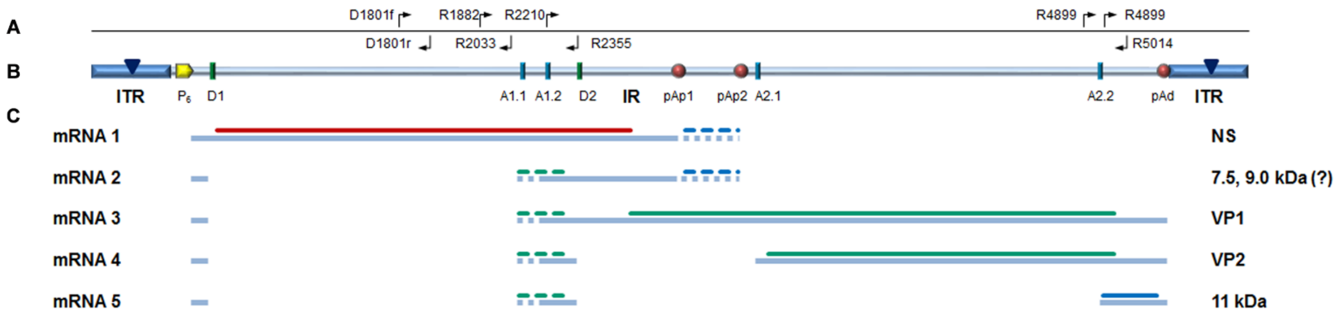

Figure S1. A. Location of primers used for qPCR and qRT-PCR analysis (Table 1). **B.** Map of B19V genome. ITR: inverted terminal repeats (▼, site of dyad symmetry). IR: internal region and relevant cis-acting functional sites (P6, promoter; pAp1, pAp2, proximal cleavage-polyadenylation sites; pAd, distal cleavage-polyadenylation site; D1, D2, splice donor sites; A1.1, A1.2, A2.1, A2.2, splice acceptor sites). Coding sequences for viral NS, VP and smaller non-structural proteins are aligned to map. **C.** Map of B19V derived transcripts; simplified transcription map, indicating the five major classes of mRNAs (mRNA 1-5), with alternative splicing forms (dashed lines) and related coding potential. Adapted from ref [10].

Table S1. Quantitation of viral nucleic acids

A. Functional competence of B19V inserts. Quantitation of viral nucleic acids in UT7/EpoS1 cells, transfected with CH10 and CH10-pAs1 derived inserts.

	DNA		RNA (total)		RNA (NS)	
Insert	8 hpt	24 hpt	8 hpt	24 hpt	8 hpt	24 hpt
CH10	6.41E+06	1.73E+06	2.50E+04	6.64E+04	4.27E+02	3.99E+02
CH10-pAs1	7.39E+06	1.88E+06	7.53E+04	5.01E+05	3.97E+02	7.02E+02
CI0	9.52E+06	8.06E+05	2.34E+04	7.21E+04	1.24E+02	2.31E+02
CI0-pAs1	9.07E+06	2.53E+06	5.90E+04	2.83E+05	3.87E+02	1.72E+02
CJ0	6.21E+06	1.30E+06	5.81E+03	2.94E+04	7.63E+01	2.46E+02
CJ0-pAs1	1.10E+07	1.92E+06	7.90E+03	1.35E+04	1.17E+02	9.00E+01

Amounts of target copies (viral DNA, total RNA, NS1 mRNA), normalized to 10^5 cells, at 8 and 24 hpt. Mean of duplicate determinations for two different experiments.

B. Functional complementation of B19V inserts. Quantitation of Viral nucleic acids in UT7/EpoS1 cells, transfected/cotransfected with CH10, CH10-pAs1 and CH10-A1.1/2 inserts.

Insert(s)	DNA total	RNA (total)	RNA (NS)	RNA (pAd)	RNA (VP)
CH10	1.38E+06	3.24E+05	1.95E+04	9.24E+04	5.23E+04
CH10-pAs1	2.33E+06	1.54E+06	8.09E+04	1.00E+00	1.00E+00
CH10-A1.1	1.50E+06	1.98E+03	1.00E+00	1.00E+00	1.00E+00
CH10-A1.2	1.00E+06	4.60E+03	1.00E+00	1.00E+00	1.00E+00
pAs1 + A1.1	1.78E+06	6.21E+05	5.81E+04	1.29E+04	7.97E+03
pAs1 + A1.2	1.52E+06	1.37E+05	3.37E+03	2.40E+04	8.89E+03

Amounts of target copies (viral DNA, total RNA, NS1 mRNA, pAd cleaved RNA, VP RNA), normalized to 10^5 cells, at 24 hpt. Mean of duplicate determinations for two different experiments.
